# Variation of sexual dimorphism and asymmetry in disease expression of inflammatory arthritis among laboratory mouse models with different genomic backgrounds

**DOI:** 10.1186/s42826-023-00185-0

**Published:** 2023-12-20

**Authors:** Wei Dong, Cheng Tian, Z. Galvin Li, David Brand, Yanhong Cao, Xiaoyun Liu, Jiamin Ma, Andy Chai, Linda K. Myers, Jian Yan, Karen Hasty, John Stuart, Yan Jiao, Weikuan Gu, Xiaojun Cai

**Affiliations:** 1 Department of Orthopaedic Surgery and Biomedical Engineering, University of Tennessee Health Science Center, Memphis, TN 38163 USA; 2 Department of Gynecology, Harbin Medical University Cancer Hospital, Harbin, 150001 Heilongjiang China; 3 St. Jude Children’s Research Hospital, Memphis, TN USA; 4 Research Service, Veterans Affairs Medical Center, 1030 Jefferson Avenue, Memphis, TN 38104 USA; 5 Department of Medicine, University of Tennessee Health Science Center, Memphis, TN 38163 USA; 6 Institute of Kaschin-Beck Disease, Center for Endemic Disease Control, Chinese Center for Disease Control and Prevention, Key Laboratory of Etiologic Epidemiology, Education Bureau of Heilongjiang Province & Ministry of Health (23618104), Harbin Medical University, Harbin, 150081 China; 7 Center for Clinical Precision Medication, The First Affiliated Hospital, Guangdong Pharmaceutical University, Guangzhou, 510006 China; 8 Clinical Pharmacy (School of Integrative Pharmacy, Institute of Integrative Pharmaceutical Research), Guangdong Pharmaceutical University, Guangzhou, 510006 China; 9 Heilongjiang Academy of Sciences of Traditional Chinese Medicine, No. 72 Xiangan Street, Xiangfang District, Harbin, 150036 China

**Keywords:** Inflammation, Sex difference, Rheumatoid arthritis, Severity, Mouse model

## Abstract

Sex difference has shown in the arthritis diseases in human population and animal models. We investigate how the sex and symmetry vary among mouse models with different genomic backgrounds. Disease data of sex and limbs accumulated in the past more than two decades from four unique populations of murine arthritis models were analyzed. They are (1) interleukin-1 receptor antagonist (IL-1ra) deficient mice under Balb/c background (Balb/c KO); (2) Mice with collagen II induced arthritis under DBA/1 background; (3) Mice with collagen II induced arthritis under C57BL/6 (B6) background and (4) A F2 generation population created by Balb/c KO X DBA/1 KO. Our data shows that there is a great variation in sexual dimorphism for arthritis incidence and severity of arthritis in mice harboring specific genetic modifications. For a F2 population, the incidence of arthritis was 57.1% in female mice and 75.6% in male mice. There was a difference in severity related to sex in two populations: B6.DR1/ B6.DR4 (*P* < 0.001) and F2 (*P* = 0.023) There was no difference Balb/c parental strain or in collagen-induced arthritis (CIA) in DBA/1 mice. Among these populations, the right hindlimbs are significantly higher than the scores for the left hindlimbs in males (*P* < 0.05). However, when examining disease expression using the collagen induced arthritis model with DBA/1 mice, sex-dimorphism did not reach statistical significance, while left hindlimbs showed a tendency toward greater disease expression over the right. Sexual dimorphism in disease expression in mouse models is strain and genomic background dependent. It sets an alarm that potential variation in sexual dimorphism among different racial and ethnic groups in human populations may exist. It is important to not only include both sexes and but also pay attention to possible variations caused by disease expression and response to treatment in all the studies of arthritis in animal models and human populations.

**Supplementary Information:**

The online version contains supplementary material available at 10.1186/s42826-023-00185-0.

## Background

Sexual dimorphism of disease expression in Rheumatoid Arthritis (RA) and Osteoarthritis (OA) has been widely reported [1, 2]. RA is typically more prevalent in women, with a female to male ratio of 3:1[3]. OA is the most common form of arthritis. The prevalence of symptomatic knee of OA was 38.5% higher among females [4].

Although sex differences may play a complex role in the expression of autoimmune disease, a good understanding of the mechanism(s) underpinning this differential expression is still lacking [5]. The reasons for the apparent lack of systematic analysis are not clear. Many studies use either female or male animals alone. Female mice are often chosen because in some mouse strains housing multiple male mice in a single cage results in aggression during the establishment of a social hierarchy. Housing males and females in a single cage is problematic at best.

Clinically, while arthritis does not always exhibit a bilaterally symmetrical expression, there is no reported evidence for left/right limb bias. There were reports on the paw preferences in mice with a complexity of environmental and genetic influence [6, 7]. It has not been reported any left/right bias in disease onset or where the potential paw usage affects disease severity or incidence in arthritis.

We hypothesize that sexual dimorphism of arthritis expression can be influenced by multiple genetic and environmental factors and that this may result in variances between individual mouse strains. Thus, the sex difference is difficult to predict and must be included in all factors in the experiments. This study used a dataset covering a very large number of positive control mice used in other studies in order to examine the influence of sex and left/right bias in multiple mouse strains.

## Main text

### Data sources

In this study, four distinct populations of murine arthritis models were examined. All four studies were approved by the institutional animal care and use committee (IACUC) boards of the University of Tennessee Health Science Center and Memphis VA Medical Center at the time of the research. Each strain has been investigated for diseases in the authors' laboratories, and the corresponding protocols and treatments have been previously reported [8–15].

#### Balb/c IL-1rn knockout mouse

Spontaneous arthritis disease occurs in interleukin-1 receptor antagonist (IL-1ra) deficient mice where the IL-1rn gene is knocked out]. IL-1ra deficient mice develop arthritis under the susceptible strain Balb/c IL-1rn^−/−^ (Balb/c KO) but not under the resistant strain DBA/1 IL-1rn^−/−^ (DBA/1 KO) [9]. After four months, arthritis can be observed in the joints and paws of Balb/c KO mice.

#### Collagen induced B6.DR1/ B6.DR4 mouse

B6.DR1/ B6.DR4 mouse is a “humanized” mouse model in which I-A˚ (mouse class II-null) C57BL/6 mice were provided with a transgene encoding a chimeric form of mouse/human RA/PD susceptibility allele HLA-DRβ1(*0101 / *0401) [10]. The only difference between B6.DR1 and B6.DR4 is the alleles expressed as transgenes in each line. Because vendor-specific enteric flora can have dramatic effects on the expression of arthritis in C57BL/6 (B6) mice [11], care must be observed in choosing the source of WT B6 mice. We have found that expression of specific human RA susceptibility HLA alleles for DR1 DR4 has resulted in a reliable expression of both CIA severity and incidence in C57BL/6 wild type mice [10] independent of the nature (human, fowl or bovine) of the type II collagen used to induce the disease.

#### Collagen induced DBA/1 mouse

DBA/1 mice are the most widely used mouse strain in the collagen-induced arthritis (CIA) model. It has served as the “gold standard” strain for CIA, which is an experimental autoimmune disease by immunization with heterologous type II collagen emulsified in complete Freund’s adjuvant [12–14].

#### F2 generation crossed by Balb/c KO and DBA/1 KO

F1 was produced by crossing Balb/c KO mice with DBA/1 KO mice, both of which were IL-1rn knockout. Then the F2 generation was produced by F1 self-breeding [15]. The F2 generation had the phenomenon of trait segregation where arthritis could be observed with great variation in some mice while the other mice are healthy.

### Disease scoring

All mice in these studies have following the same scoring method. Each limb was graded on a scale of 0–4 for degree of redness and swelling (0 = no evidence of erythema and swelling, 1 = mild redness and swelling of joint and ankle, 2 = definite swelling, 3 = severe swelling of entire limb, and 4 = limb burned out and deformed) [8–15]. As such, when the scores of 4 limbs was calculated, the maximum 16 points is for an individual mouse.

#### Data collection and analytic methods

Data from individual strains of mice were gathered from different labs. The arthritis severities were scored by different technicians, but all using the same method [14]. The hindlimb was graded on a scale of 0–4 for degree of redness and swelling, with 0 being no evidence of erythema and swelling and 4 being the limb burnt out and deformed [16]. In this research, we analyzed 47 Balb/c Il1rn knockout mice and 343 F2 generation mice (mice used in our previous studies), 333 B6.DR1 mice and 57 B6.DR4 mice (Data from Dr. Brand’s lab), and 67 DBA/1 mice (Data from Dr. Brand’s and Dr. Myers’s lab). The Student t-test was performed to make comparisons between groups, and *P* < 0.05 represents a significant difference.

### Sex difference in the arthritis severity of mice

**Fig. 1:**
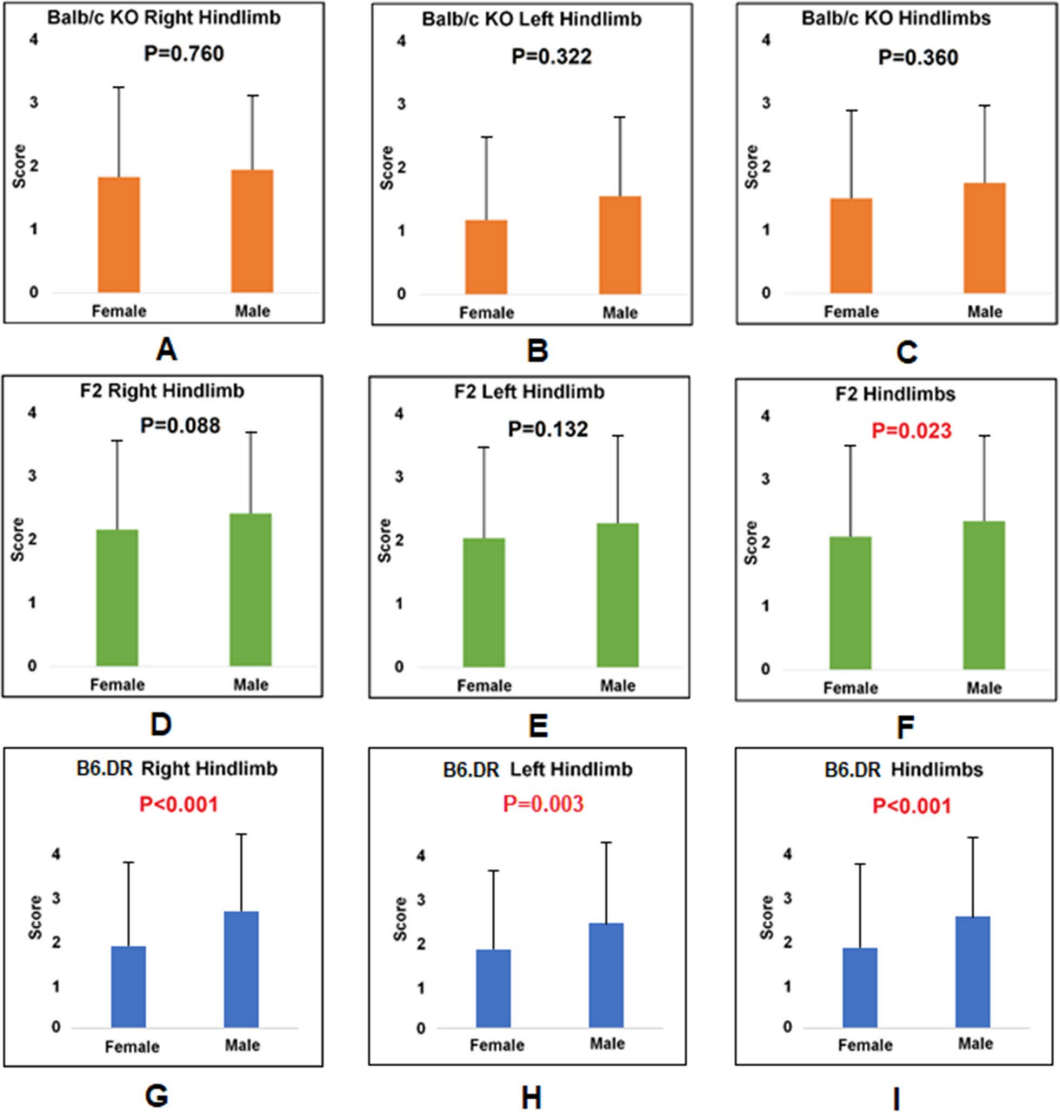
Sex differences in different arthritis mouse strains. A/B/C: Balb/c KO mice. D/E/F: F2 generation crossed by Balb/c KO and DBA/1 KO. G/H/I: B6.DR mice. Error bars indicate the standard variations of the disease scores

**Fig. 2:**
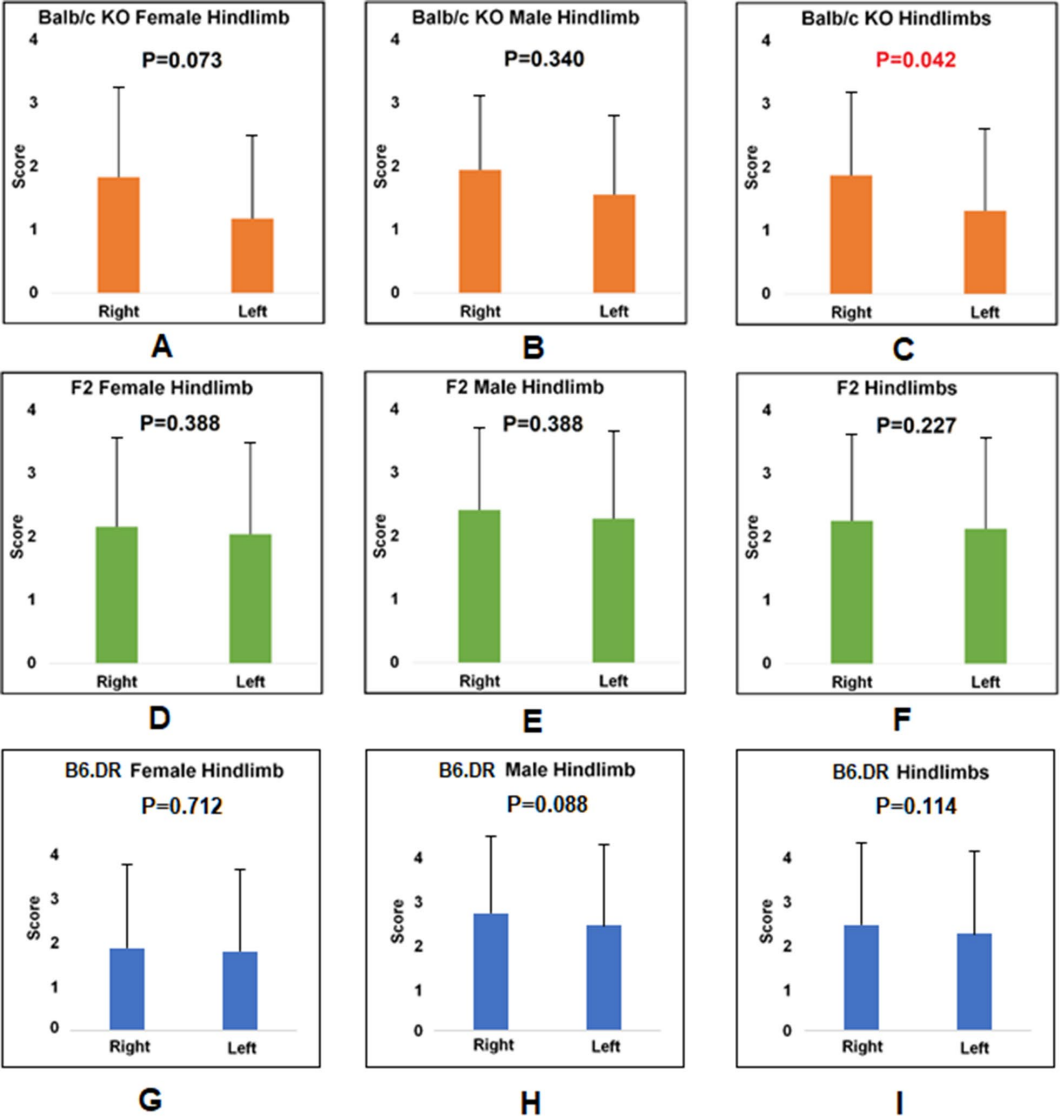
Side differences in different arthritis mouse strains. A/B/C: Balb/c KO mice. D/E/F: F2 (Balb/c KO × DBA/1 KO) generation. G/H/I: B6.DR mice. Error bars indicate the standard variations of the disease scores

We first compared the sex difference in arthritis scores (Fig. 1). For this, the severity scores for the male and female mice were compared across the right hindlimb only, the left hindlimb only, and all hindlimbs. Next, we compared the difference in arthritis scores between the left and right hindlimbs (Fig. 2). The severity scores for the left and right hindlimb were compared across female mice, male mice, and all mice (Additional file 1: Fig. S1).

As shown in Fig. 1, there is a significant difference in arthritis severity scores between male and female hindlimbs in the F2 generation (*P* = 0.023). B6.DR mice also showed an even more significant difference in arthritis severity scores between males and females (*P* < 0.001). In both the B6.DR mice and F2 groups, it appears that males in general have higher arthritis severity scores than their female counterparts. In the Balb/c KO groups, this trend is present, but a significant difference cannot be observed.

### Side difference in the arthritis severity of mice

In Fig. 2, the results show that the Balb/c KO, F2 generation, and B6.DR groups all displayed the pattern of having a higher severity score in the right hindlimb than the left hindlimb. However, only in Balb/c ko strains does this difference reach statistical significance (*P* = 0.042).

### Sex difference and side difference of RA mice (Balb/c KO, F2 generation, and B6. DR)

**Fig. 3:**
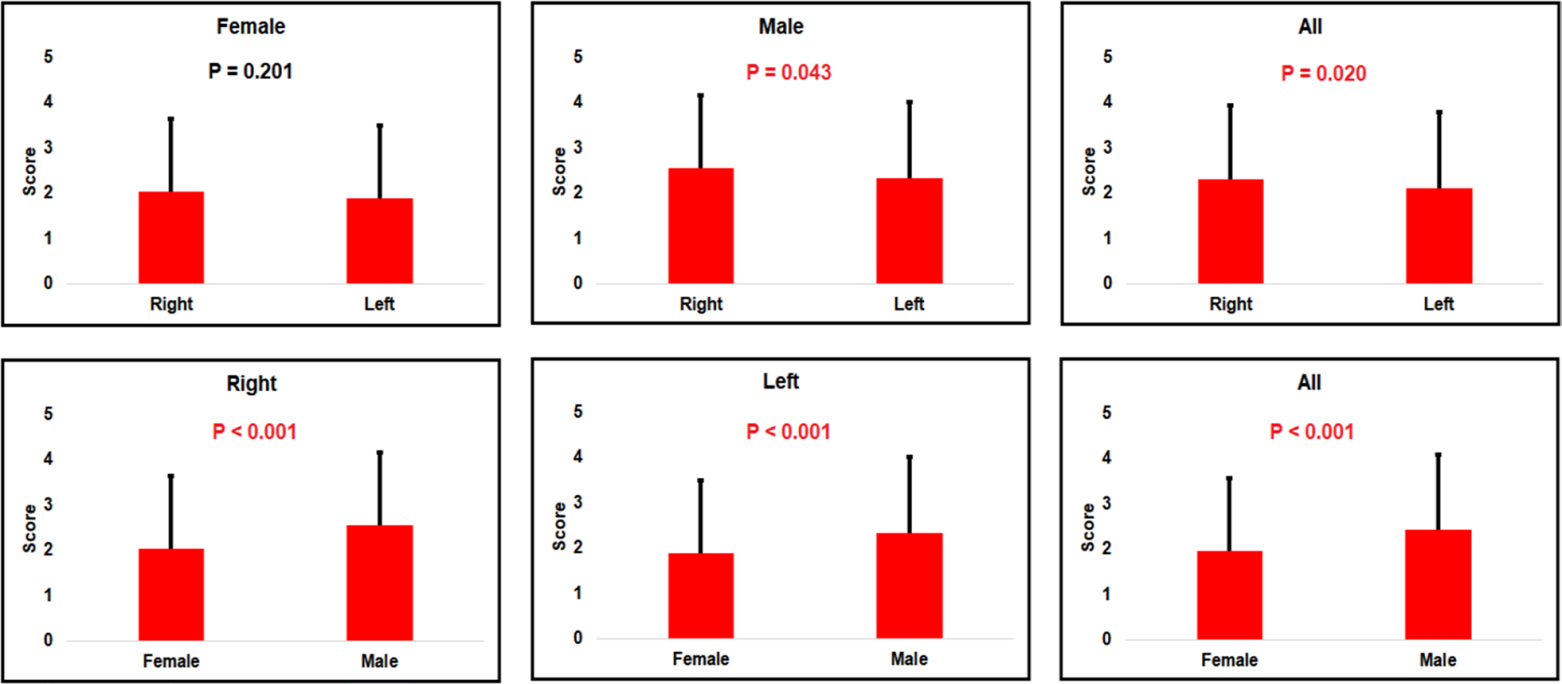
Side and sex difference in arthritis severity in mice. Error bars indicate the standard variations of the disease scores

We combined the Balb/c IL-1ra knockout strain’s, the F2 generation’s, and the B6.DR strain’s data together to form a composite dataset with a total number of 780 mice. As shown in Fig. 3, the results indicated that the scores for the right hindlimbs are significantly higher than the scores for the left hindlimbs in males (*P* < 0.05). Furthermore, arthritis in male mice is significantly more severe than in females (*P* < 0.001).

### Sex difference and side difference in Collagen induced DBA/1 mice

**Fig. 4:**
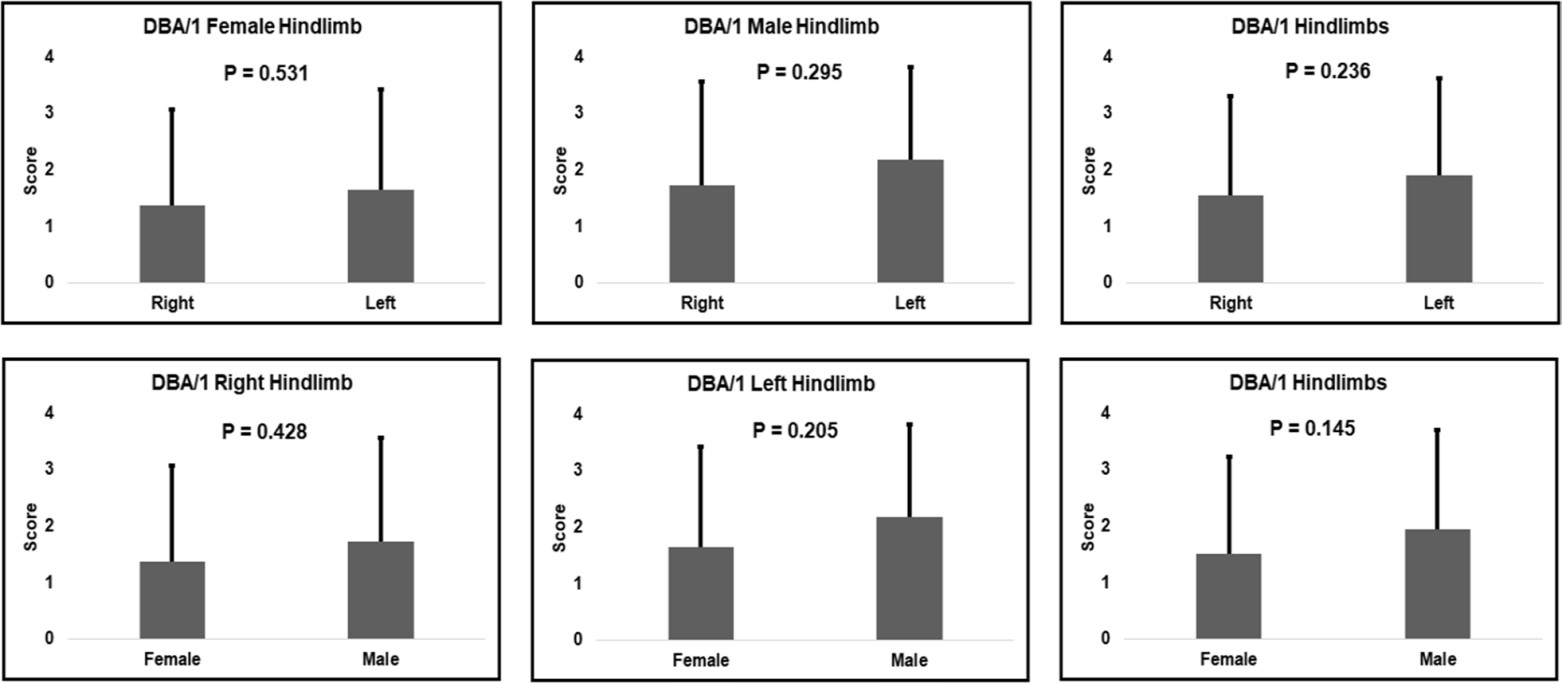
Left/right bias and sexual dimorphism in CIA disease severity in DBA/1 mice. Error bars indicate the standard variations of the disease scores

In collagen induced DBA/1 mice, there is no significant difference in arthritis severity scores with respect to sex or bias toward a specific side. However, unlike the other groups, the pattern of side difference is reversed—our data showed that severity scores were, in general, higher for the left hindlimb than the right hindlimb (Fig. 4). This indicates that the phenotype of arthritis in different mouse strains might be separately considered. This data is based on 67 collagen induced DBA/1 mice. More mice should be collected to verify this result in the future.

### Arthritis incidence in F2 generation crossed by Balb/c-/- and DBA/1-/- mice

A total of 542 mice of the F2 generation were observed by technicians as having arthritis or healthy, and the results are displayed in Table 1. The incidence of arthritis is 57.1% in female mice and 75.6% in male mice. Statistical analysis indicated that the incidence of arthritis is significantly higher in male mice than in female mice (*P* = 0.00003).

**Table 1:** Arthritis incidence in F2 generation crossed by Balb/c KO and DBA/1 KO mice

F2 generation	Female	Male	X^2^	P
Arthritis	207	136	17.466	**0.00003**
Healthy	155	44		
Total number	362	180	542	
Incidence	57.18%	75.56%		

### Disease onset day between left and right legs

In order to see whether the sexual dimorphism and asymmetry of the disease is influenced by disease onset day in the legs, we examined the first day of disease onset between left and right legs. Our data indicated that, unlike the sex difference, there was no difference between left and right legs on the first disease onset (Table 2). These differences did not appear in either female or male mice.

**Table 2:** Disease onset day between legs in different mouse strains

Strains	Sex	Left	Right	Both
Balb/c-/-	Female	9	5	3
	Male	10	4	3
B6.DR1	Female	35	34	12
	Male	91	106	21
F2	Female	23	27	20
	Male	13	11	10
DBA/1	Female	3	14	4
	Male	8	6	2

As shown above, our study highlights the importance of including both sexes and paying attention to the influence of disease phenotypes, along with multiple other factors, in the study of arthritis diseases using a mouse model. When studying RA animal models, it is crucial to differentiate between the effects of sex differences among different mouse strains on the results of experimental arthritis. In this article, we compared the sex differences in the arthritis severity of various types of arthritis mouse models, including the mutate models of IL-1rn mutation under different genomic backgrounds, namely Balb/c and DBA/1, the CIA model, a “humanized” mouse model in a different background, namely C57BL/6, and the F2 generation of the genetic arthritis mouse created between the knockout model of Balb/c and DBA/1. We scored and analyzed more than 700 mice and concluded that the severity and incidence of arthritis differ for both sexes in mice. Among the groups we examined, the severity of arthritis in male mice was higher than that in females. Many studies have confirmed the role of sex hormones in RA [16]. However, report on the sexual dimorphism in rodent model is mixed. A recent review by Delay et al. summarized the arthritis score in the complete Freud’s adjuvant (CFA), the collagen induced arthritis (CIA), the collagen-antibody-induced arthritis (CAIA) and K/BxN (sera from KRN-NOD transgenic mice) passive transfer models [17]. It reported sex ratio of rats in several studies with controversial results. Although females exhibited a higher arthritis score in CFA rats [18] and in rats with collagen-induced arthritis, no difference was observed in arthritis development between sexes in the CAIA [19, 20] and K/BxN passive transfer mouse model [21], In summary, it seems that the degree of sex difference in arthritis scores varies for different species and different strains.

In comparison with the sex difference, the Balb/c parental strain or CIA in DBA/1 mice, the right hindlimb scores are higher than the left hindlimb scores in males, but with no significant level (Fig. 2). When analyzed together with data from all three sets of animal models, the score in right hindlimb is higher than that in left hindlimb with *P* = 0.043 (Less than 0.01 but more than 0.05) (Fig. 3). Hence, there is a potential possibility that the score in right hindlimb is higher than the left one in male mice. Such a difference may be caused by multiple reasons. One possibility is the activities of males in the cage, thus, the right turns or the left turns when running in the cage. The other reason may be the habit of drinking water. Whether the males is leaning on the right or left when drinking water. Also, genetic and developmental differences between right and left hindlimb can not be ruled out. As such, to determine whether and why the score is different between right and left hindlimbs in males, further investigation on this aspect is needed.

For the F2 generation, the arthritis incidence in females is much lower than that in males. In contrast, however, for humans, the ratio of the occurrence of RA in women to that in men is typically around 3:1 [22]. This discrepancy may be due to the species difference and that the F2 generation of mice is a population with a wide genetic segregation from the Balb/c and DBA/1 backgrounds. However, it is still essential to explore the incidence difference between humans and mice.

The complicated interaction between genetic backgrounds and the environmental factors may influence the sex difference in the disease development. Among these statistical analyses, the results we obtained for DBA/1 mice were different from the other groups. This may be because of the different genetic background. Mouse strains with different genetic backgrounds showed different phenotypes in RA. When IL1rn was knocked out, arthritis did not develop in DBA/1 mice but expression continued in Balb/c mice [9]. Another example of strain differences in response to CIA is that chicken type II collagen can serve as an immunogen to induced arthritis in DBA/1 mice but it is ineffective in Balb/c mice. This may be due to differences in binding or presentation of the specific collagen moiety by the mouse class II MHC molecule I-A^q^ (DBA/1) vs. I-A^d^ for Balb/c. These example shows that in the same environment condition, different genotype or mouse strain may have different disease dimorphisms. Also, the same genotype under different environmental conditions may result in different dimorphism too. Thus, the combination of genetic factors and the environmental factors leads to the sexual dimorphism among different mouse models. These phenomena also set alarm for the animal studies in which one model may be suitable for a population of a certain genotypes or ethnic groups.

We also showed that the experimental arthritis expression has exhibited a left/right bias, resulting in greater expression in the right hindlimb than in the left hindlimb in mice. Some clinical studies [23, 24] have suggested that there is no side predilection difference between left hand and right hand in human RA patients. While more data should be collected to validate the results in mice, this data sets an alarm on the potential variations between sex in disease expression and response to therapeutic treatment among different racial and ethnic groups in human populations.

In this research, we collected the scores of arthritis mice used in previous studies from different labs. These incidence and severity data were collected prior to this analysis. The acquisition of this data was performed in a double-blind fashion and statistics were applied without regard to the results of the previous experiments. There was no prior knowledge that the data would be used to analyze these differences before scoring, so bias can be eliminated to the greatest extent. Additionally, the results in different groups were similar. We believe these results to be both reliable and significant, and hope they can contribute to a better understanding of the data interpretation for mouse models of RA.

The sex difference in the disease incidence of the mouse models in our study shows that it is different from humans, in which the women have a higher prevalence of arthritis than men. This data remined us that the mouse is not the human, despite the 99% same in genome between the mouse and the human. Any data from the mouse model needs to be tested and confirmed by human population or other models. In the other hand, mouse model is still useful for the study of human arthritis due to the fact that it can be manipulated genetically and environmentally to obtain comparable and reliable data, that it can be utilized in the study of molecular pathways and select targets for the therapeutic application. Thus, mouse models are useful while caution should be taken. Furthermore, both sexes should be included in the studies.

## Conclusions

This study is significant as it systematically investigates the sexual dimorphism and asymmetry in disease expression in arthritis among different mouse models. It suggests that sex differences, as well as environmental and treatment influences, may contribute to variations among experimental mouse models and their potential translation to the human population. Therefore, sex differences should be considered a potential influencing factor in future studies using mouse models as well as in human populations, particularly in studies of disease phenotype, mechanisms, and drug testing.

### Supplementary Information

**Additional file 1: Figure 1**. Graphic demonstration of disease score distribution between left and right hind legs among different populations.

## Data Availability

All datasets presented in this study are either included in the article or in public databases which have been stated in the article.

## Abbreviations

Balb/c KO: Balb/c IL-1rn^−^^/−^
B6: C57BL/6
CIA: Collagen-induced arthritis
DBA/1 KO: DBA/1 IL-1rn^−^^/^^−^
IACUC: Institutional animal care and use committee
OA: Osteoarthritis
RA: Rheumatoid arthritis
