# Supplementary material for: Variation of sexual dimorphism and asymmetry in disease expression of inflammatory arthritis among laboratory mouse models with different genomic backgrounds
Source: Lab Anim Res. 2023 Dec 20;39:35. doi: 10.1186/s42826-023-00185-0 (PMC10731690; doi:10.1186/s42826-023-00185-0)
Supplement: Supplementary file 1 — Additional file 1: Figure 1. Graphic demonstration of disease score distribution between left and right hind legs among different populations. [file 42826_2023_185_MOESM1_ESM.docx]

Supplementary Figure 1. Graphic demonstration of disease score distribution between left and right hind legs among different populations.

A. In B6.DR1.DBA population

B. In Balb/c knockout mice

C. In F2 generation
